# Synergistic Effect of Aluminum Nitride and Carbon Nanotube-Reinforced Silicon Rubber Nanocomposites

**DOI:** 10.3390/molecules29122864

**Published:** 2024-06-16

**Authors:** Jie Gao, Houhua Xiong, Xiaobing Han, Fei An, Tao Chen

**Affiliations:** Hubei Key Laboratory of Radiation Chemistry and Functional Materials, School of Nuclear Technology and Chemistry & Biology, Hubei University of Science and Technology, Xianning 437100, China; gaojie2019@hbust.edu.cn (J.G.); xionghouhua@hbust.edu.cn (H.X.); an28907363@163.com (F.A.)

**Keywords:** synergistic effect, aluminum nitride, carbon nanotube, silicon rubber, properties

## Abstract

Constructing a synergistic effect with different structural fillers is an important strategy for improving the comprehensive properties of polymeric composites. To improve the comprehensive properties of two-component additive liquid silicon rubber (SR) materials used in electronics packaging, the synergistic effect of granular aluminum nitride (AlN) and tubular carbon nanotube (CNT)-reinforced SR nanocomposites was investigated. AlN/CNT/SR composites with different AlN/CNT ratios were fabricated with two-component additive liquid SR via the thermal curing technique, and the influence of AlN/CNT hybrid fillers on the hardness, strength, elongation at break, surface resistivity, thermal conductivity, and thermal decomposition was investigated in detail. With the incorporation of AlN/CNT hybrid fillers, the comprehensive properties of the obtained AlN/CNT/SR composites are better than those of the AlN/SR and CNT/SR composites. The synergistic thermal conductive mechanism of AlN/CNT hybrid fillers was proposed and demonstrated with the fractural surface morphology of the obtained composites. The obtained AlN/CNT/SR composites show promising applications in electronic packaging, where necessary mechanical strength, electrical insulating, thermal conductivity, and thermal stable materials are needed.

## 1. Introduction

Due to its flexibility, electrical insulation, anti-aging, high- and low-temperature resistance, barrier, small linear shrinkage, dimensional stability, and curing without by-products, silicon rubber (SR) has been widely applied in composite and functional materials, such as flame retardant and superhydrophobic coating, flexible and wearable devices, medical dressings, and stents [1,2,3,4,5,6]. Though SR possesses many features, it suffers from low mechanical properties, low thermal conductivity and ablation resistance, low electrical conductivity, and low surface energy. Thus, many kinds of modifications were developed to enhance the comprehensive performance of pure silicone rubber.

To improve these properties of pure SR materials, many filler-filled SR composites were developed [7,8,9,10,11]. Montmorillonite (MMT) was incorporated into the SR to improve the mechanical and tribological properties, and SR composites filled with 2 wt.% MMT exhibit improved performance [12], showing 6% and 10% improvement in tear strength and tensile strength. To improve the thermal conductivity, modified boron nitride (BN)/SR composites were fabricated based on an “in-situ welding” strategy [13]. BN/SR composites of very low thermal resistance (˂70 Kmm^2^/W) and high thermal conductivity (15.4 W/m·K) were obtained, with relatively low BN loading (15 wt.%). To improve the low char yield, well-dispersed phenolic resin/SR composites with high ablation resistance were developed [14]. Compared with raw SR, the obtained composites exhibit 44.19% and 17.81% reduction of mass ablation rates, respectively. To improve the low electrical conductivity, modified carbon fiber (CF)-filled SR composites were investigated [15]. The volume resistivity at the perpendicular direction of the obtained CF/SR composites was 6.8 times higher than the orientation direction. Though great progress was made in SR-based materials, the improvement of comprehensive properties of SR materials cannot always be achieved with a single filler [10].

To address the deficiency of single filler-incorporated polymer composites, a synergistic effect based on hybrid fillers was developed for the construction of high-performance composites [16,17,18,19,20,21,22]. The hybrid filler system can accelerate the construction of a continuous filler structure and upgrade the dispersion/exfoliation of each filler. Obvious synergistic effects were found in hybrid filler-incorporated polymer composites with graphene/carbon black [23], carbon nanotubes/carbon black [24], graphite/carbon nanotubes [25], graphene/boron nitride [26], aluminum nitride/carbon nanotubes (AlN/CNTs) [27], etc. Among these hybrid filler systems, AlN/CNT hybrid fillers have been widely used in the area of electronic packaging due to the high thermal conductivity, mechanical properties, and high insulating properties [28,29,30]. With the incorporation of AlN/CNT hybrid fillers, cyanate ester (CE)-based nanocomposites demonstrated dramatic improvement in comprehensive properties [31], and the obtained AlN/CNT/CE composites showed flame retardant properties, high thermal conductivity, high storage modulus, and low dielectric loss. AlN/CNT/epoxy resin (EP) nanocomposites were fabricated via casting techniques [32], and the obtained AlN/CNT/EP composites possessed a thermal conductivity as high as 0.48 W/m·K, which is two times that of raw EP. Compared with the volume resistivity of raw EP (2.5 × 10^14^ Ω·m), the surface resistivity of the AlN/CNT/EP composites showed a small decrease (1.8–2.6 × 10^12^ Ω·m), which can be applied in electronic packaging.

Synergistic effects based on hybrid fillers were also observed in silicon rubber-based materials [33,34,35]. Most of the hybrid filler’s synergistically improved SR composites were focused on the enhancement of thermal conductivity. Micro-nano hybrid fillers based on alumina (Al_2_O_3_) and CNTs were used for the fabrication of electrical packaging SR materials, which exhibit high electrical insulation and thermal conductivity [36]. The obtained Al_2_O_3_/CNT/SR composites possessed a high volume resistance of 1.323 × 10^9^ Ω·cm and a high thermal conductivity of 1.137 W/m·K. Al_2_O_3_/graphene hybrid fillers were also developed for the preparation of SR-based thermal grease. Compared with pure SR grease, the thermal conductivity enhancement reached 2553% [37]. Polydopamine modified with silver-deposited CNT and BN hybrid fillers was also reported for the thermal conductivity enhancement of SR [38,39], with the conductivity of the obtained composites improving by 4.17 times. There are few reports about the improvement in comprehensive properties with hybrid fillers. With the incorporation of Al_2_O_3_/CNT hybrid fillers, except for the improvement of thermal conductivity (0.13 to 0.26 W/m·K), the modulus and hardness of the obtained composite also improved [40]. Branched Al_2_O_3_ was prepared to synergistically reinforce SR materials with CNTs, and high thermal conductivity (1.307 W/m·K), high volume resistivity (10^15^ Ω·cm), high tensile strength (4.47 MPa), and high elongation (206.9%) were observed for the branched Al_2_O_3_/CNT/SR composites [41].

Though the AlN/CNT hybrid fillers showed obvious synergistic effects in the comprehensive property enhancement of other polymer composites, there are very few reports about these hybrid fillers incorporating silicone rubber (SR) composites, especially for comprehensive property improvement [42]. Thus, AlN/CNT hybrid fillers incorporating SR (AlN/CNT/SR) were prepared via a thermal curing technique with two-component additive liquid silicon rubber, and the synergistic effect of different AlN/CNT ratios on the Shore A hardness, tensile strength, elongation, electrical insulation, thermal conductivity, and thermal stability were investigated in detail. For comparison, AlN/SR and CNT/SR composites were also fabricated and investigated.

## 2. Results and Discussion

### 2.1. Morphology of Nanofillers

**Figure 1:**
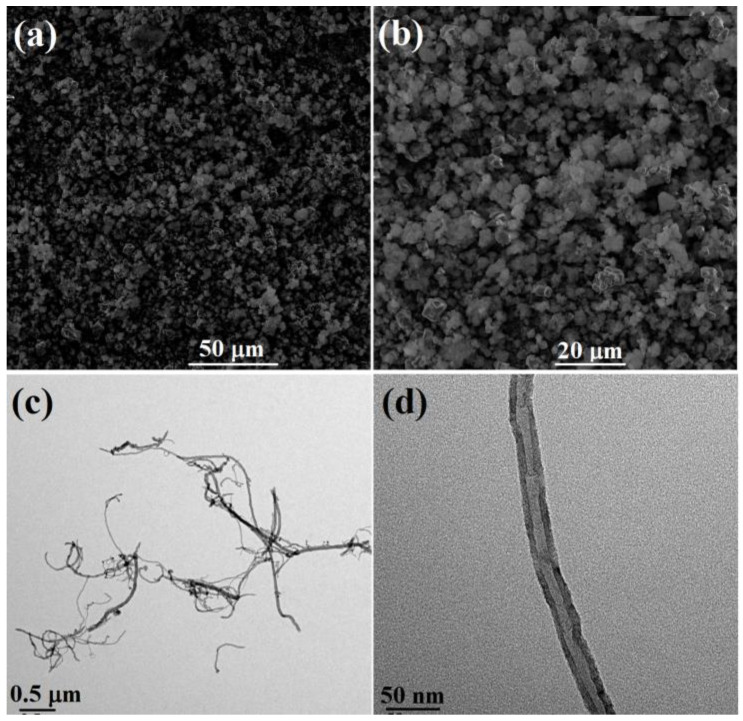
SEM images of AlN (**a**,**b**) and TEM images of CNTs (**c**,**d**).

The filler shape, diameter, length, and dispersion state are very important for constructing a continuous filler structure, which is the foundation of the synergistic effect originating from a hybrid filler [23,27,39,42]. The morphology of AlN and CNTs were characterized with SEM and TEM. As shown in Figure 1a,b, raw AlN is a uniform irregular particle with a diameter of 2–5 μm [32,41]. The CNTs had a length of about 1–5 μm (Figure 1c), and the diameter was about 30 nm (Figure 1d) [28,32]. The high aspect ratio of CNTs will benefit the formation of a continuous filler structure, and the combination of a micro–nano structure based on AlN/CNTs will form a synergistic effect for the obtained AlN/CNT/SR composite [36].

### 2.2. Mechanical Properties of AlN/CNT/SR Nanocomposites

#### 2.2.1. Hardness

**Figure 2:**
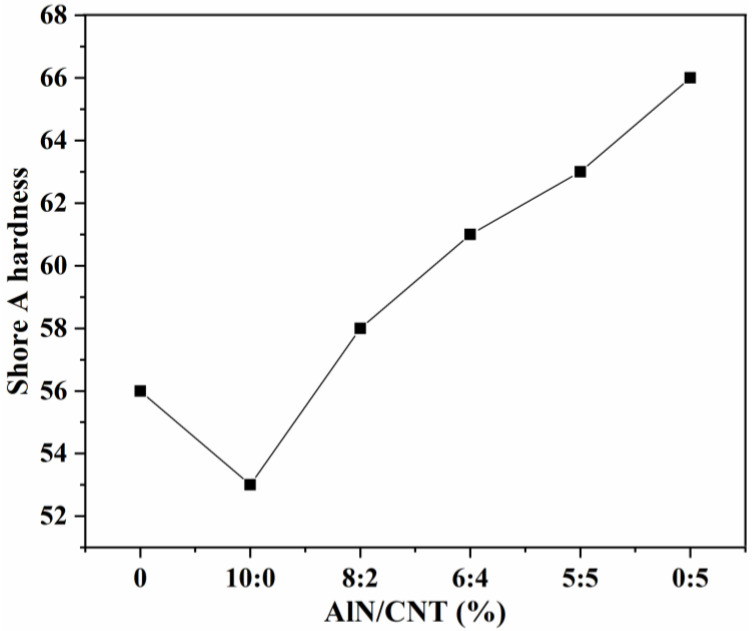
The hardness of the AlN/CNT/SR nanocomposites.

Till now, hardness has been the most widely used mechanical test to evaluate the properties of rubbers in the industrial field. The purpose of a hardness test is to evaluate the rubber cross-linking degree between the rubber matrix and different mineral fillers. The most common indentation test of SR-based materials is the Shore A hardness test, which provides information about the puncture resistance of an SR encapsulant in the work condition. The Shore A hardness of the AlN/SR, CNT/SR, and AlN/CNT/SR composites with different AlN/CNT hybrid filler ratios are presented in Figure 2. Except for the AlN/SR composite, all of the obtained composites had a higher value of Shore A hardness than that of pure SR. The hardness of pure SR was 56, and with the incorporation of a single component of AlN particles, the hardness decreased to 53, which was due to the plasticizing effect of micron-sized granular AlN under a low loading percentage [17,43]. With the incorporation of a single component of CNT, the highest hardness of 66 was observed for the obtained CNT/SR composites. This can be ascribed to the entanglement effect between tubular CNTs with SR chains, which can inhibit the slipping of SR chains [40,44,45]. Therefore, for the AlN/CNT hybrid filler-incorporated SR composites, the hardness increased from 58 to 63, while the CNT loading percentage increased from 2 wt.% to 5 wt.%.

#### 2.2.2. Tensile Strength and Elongation

**Figure 3:**
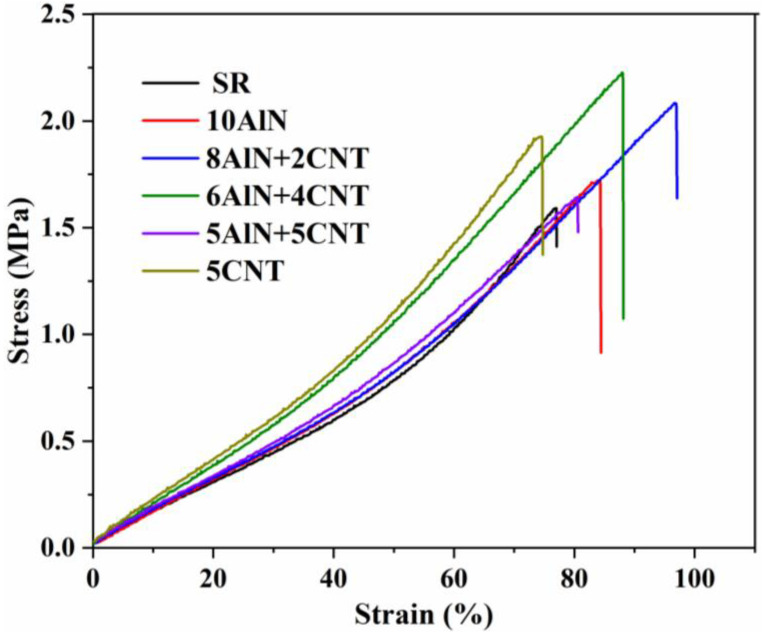
Stress–strain curves of AlN/CNT/SR nanocomposites.

Tensile strength and elongation are the basic mechanical properties of electronic packaging materials, which can reveal the maximum loading and deformation of the obtained materials. Tensile testing of the obtained AlN/SR, CNT/SR, and AlN/CNT/SR composites with different AlN/CNT hybrid filler ratios were conducted, and the stress–strain curves are presented in Figure 3. As shown in the figure, with the incorporation of a single-component filler and different AlN/CNT hybrid fillers, the tensile strength or the elongation of all the obtained composites was improved. The pure SR possessed the lowest modulus, whereas with the incorporation of single-component filler and AlN/CNT hybrid fillers, the modulus of all obtained composites increased [34,45,46,47]. A small increase in modulus was observed for the 10AlN-, 8AlN+2CNT-, and 5AlN+5CNT-incorporated composites, and an obvious increase was observed for the other two composites. The 5CNT/SR possessed the highest modulus and the lowest elongation, which is similar to the branched Al_2_O_3_-incorporated SR composite [41]. This may be caused by interfacial defects when composites are stretched. The 6AlN+4CNT/SR showed a balance between the modulus and elongation, which is similar to carbon fiber/graphene hybrid filler-incorporated polyamide [45]. This can be attributed to the CNTs having a high modulus and the synergistic effect of AlN and CNTs. As commercial fillers without chemical modification were used in this investigation, a certain degree of phase separation was observed in the filler and SR; thus, the mechanical properties of the obtained composites was lower than that of the Al_2_O_3_/CNT-incorporated SR [36,41].

**Figure 4:**
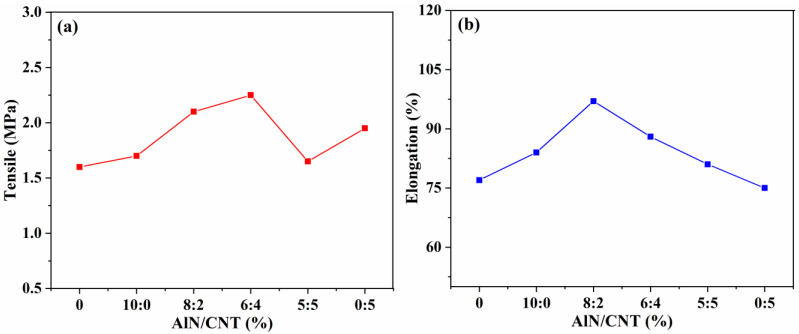
Tensile strength (**a**) and elongation at break (**b**) of AlN/CNT/SR nanocomposites.

The tensile strength and elongation at the break of the obtained AlN/CNT/SR composites are described in Figure 4. Compared to the raw SR, with the incorporation of AlN alone, the tensile strength and elongation increased 6.25% and 9.09%, respectively. With the incorporation of CNTs alone, the tensile strength increased from 1.60 to 1.95 MPa, but the elongation decreased from 77 to 75%. This reveals that the enhancement of tensile strength and elongation cannot be achieved with only one filler. All of the different AlN/CNT ratio hybrid fillers exhibited a synergistic effect toward the enhancement of both tensile strength and elongation. The highest tensile strength (2.25 MPa) was observed for the 6AlN+4CNT-filled SR composites, and the highest elongation (97%) was obtained for the 8AlN+2CNT-incorporated SR composites, which demonstrated the construction of a continuous AlN/CNT structure [45,48,49].

### 2.3. Electronic Properties of AlN/CNT/SR Nanocomposites

**Figure 5:**
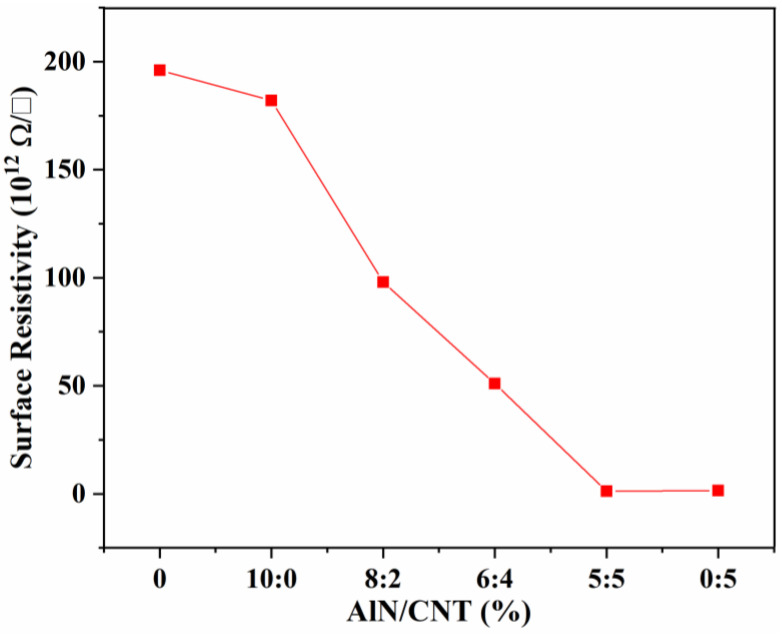
Resistivity of AlN/CNT/SR nanocomposites.

Two-component additive liquid SR is always used as an electronics packaging material, and has relatively good mechanical properties, which should also be electrically insulated [36]. As we all know, AlN particles have a high resistivity [42], while CNTs are a conductive filler. To reveal the effect of single-component filler and AlN/CNT hybrid fillers on conductivity, the surface resistivity of the obtained SR composites was investigated. As shown in Figure 5, compared with pure SR (1.96 × 10^14^ Ω/☐), with the incorporation of a single AlN filler, no obvious decrease in the surface resistivity (1.82 × 10^14^ Ω/☐) was observed, which was due to the high electrical insulation of AlN. For the AlN/CNT hybrid filler-incorporated SR composites, with the increase in CNT loading, the surface resistivity of the obtained AlN/CNT/SR composites decreased obviously, and the lowest surface resistivity (1.20 × 10^12^ Ω/☐) was observed for the 5AlN+5CNT composites. In addition, the surface resistivity of the 5CNT composites (1.50 × 10^12^ Ω/☐) was similar to that of the 5AlN+5CNT composites, which is associated with the high conductivity of CNTs [41]. Though the surface resistivity decreased obviously with the filling of CNTs, there was no conductive network constructed within the composites. The surface resistivity of all obtained composites was higher than 10^6^ Ω/☐, demonstrating that the conductivity was enhanced via the hopping mechanism with isolated filler particles [17,50,51,52].

All of the obtained composites had a surface resistivity higher than 10^12^ Ω/☐, which means that the fabricated AlN/CNT/SR composites featured electrical insulation [41,53]. This is similar to the other hybrid fillers that incorporate silicone rubber, which is suitable for application in electronic packaging materials. As reported in the literature, Al_2_O_3_/CNT [36,41] and BN/CNT [39] hybrid fillers have also been developed for the synergistic improvement of SR-based materials. All of these SR composites are electrically insulated, with a volume resistivity higher than 10^9^ Ω·cm. Though a continuous filler structure was constructed between the CNTs and the thermally conductive filler, the conductive CNTs were isolated by the electrically insulated AlN. Thus, the electrical insulation will remain, and the thermal conductivity of the AlN/CNT/SR composites will improve obviously [36,39,41].

### 2.4. Thermal Properties of AlN/CNT/SR Nanocomposites

Except for the electrical insulation, the electronics packaging materials should also have thermal management properties, including high thermal conductivity and thermal stability. Thus, the thermal conductivity and thermal stability of the AlN/CNT hybrid filler-incorporated SR composites were investigated.

#### 2.4.1. Thermal Conductivity

**Figure 6:**
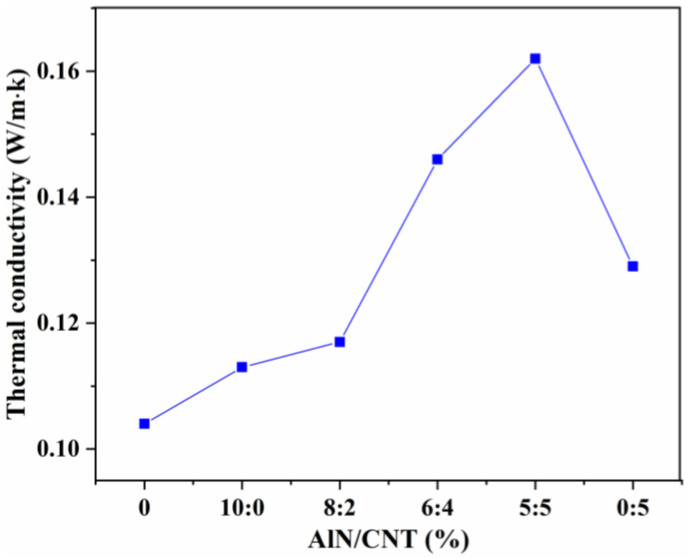
Thermal conductivity of AlN/CNT/SR nanocomposites.

As mentioned in the reported work, the intrinsic thermal conductivity of AlN and CNTs are 320 and 2800 W/m·K respectively [27,29]. To verify the synergistic effect of AlN/CNT hybrid fillers on the enhancement of thermal conductivity, the thermal conductivity of the obtained AlN/CNT/SR composites was investigated. As shown in Figure 6, the thermal conductivity of pure SR was as low as 0.104 W/m·K. With the introduction of AlN or CNTs alone, the thermal conductivity of the corresponding SR composite increased by 8.65% and 24.04%, respectively, which was due to the intrinsic thermal conductivity of CNTs being higher than that of AlN. For the AlN/CNT hybrid filler-incorporated SR composites, the thermal conductivity of the obtained AlN/CNT/SR composites increased obviously with the increase in CNT percentage, and the highest thermal conductivity (0.162 W/m·K) was observed for the 5AlN+5CNT composites. What is more, the thermal conductivity of the 5AlN+5CNT composites was 25.58% higher than that of the 5CNT composites. The highest thermal conductivity obtained with AlN/CNT hybrid fillers in this work was similar to that of Al_2_O_3_/CNT (~0.26 W/m·K) [40] but much lower than that of Al_2_O_3_@CNT (1.137 W/m·K) [36] and branched Al_2_O_3_/CNT (1.307 W/m·K) [41], which can be ascribed to the assembly or branching of the hybrid fillers used in the reported work.

**Figure 7:**
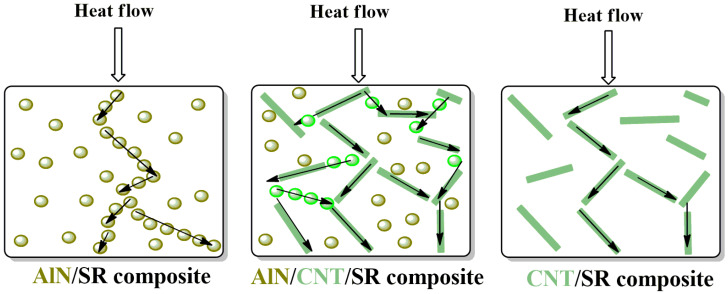
Thermal conductive mechanism illustration of different composites.

Interestingly, the thermal conductivity of the 5AlN+5CNT composites was 43.36% and 25.58% higher than that of the 10AlN composites and the 5CNT composites, which demonstrates that a continuous AlN/CNT network was constructed within the obtained AlN/CNT/SR composites and the thermal conductivity was enhanced by the synergistic effect of the AlN/CNT hybrid fillers [36,41,54]. The thermal conductive mechanism of the AlN/SR, AlN/CNT/SR, and CNT/SR composites is illustrated in Figure 7 based on the results of the fillers’ morphology and the composites’ thermal conductivity. As the AlN filler is a particle filler, high loading was needed to form a conductive network. Compared to the granular AlN, the CNTs possessed a high aspect ratio, which easily formed a conductive network. Thus, the tubular CNTs had a higher improvement efficiency than the granular AlN. The formation of a continuous filler structure is very important for enhancing the thermal conductivity, as it can reduce the thermal resistance and accelerate the transport of phonons [31,37,40]. For the AlN/CNT hybrid filler synergistically improved SR materials, except for the continuous CNT network, the isolated AlN particles can also be joined by CNTs, and thus more network structures can be formed [32,36].

**Figure 8:**
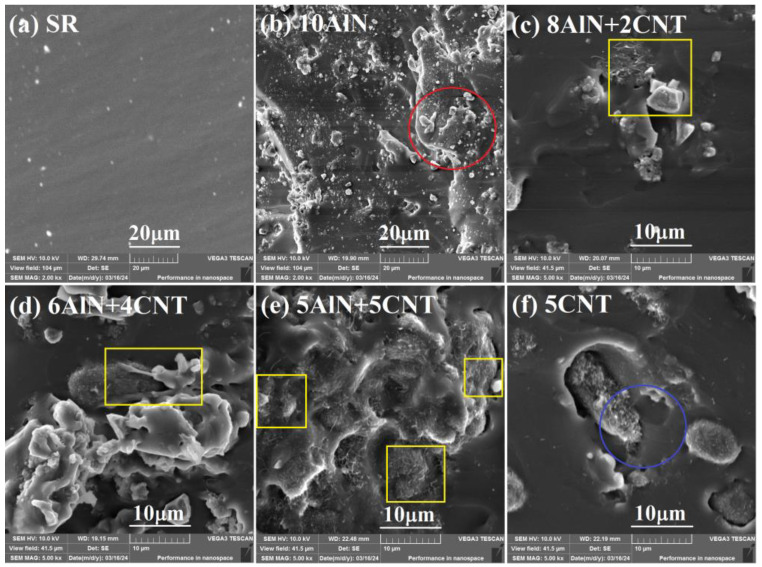
SEM images of the (**a**) SR, (**b**) AlN/SR (AlN particles were observed in the red circle), (**c**–**e**) AlN/CNT/SR (AlN/CNT hybrid fillers were presented in the yellow box), and (**f**) CNT/SR (CNT was appeared in the blue circle) nanocomposites.

To demonstrate the above proposed thermal conductive mechanism of the AlN/CNT/SR composites, the dispersion state of the AlN, CNT, and AlN/CNT fillers in the SR matrix was provided (Figure 8). As shown in Figure 8a, as no filler was incorporated, the fractural surface of pure SR was relatively flat and smooth. All of the composites showed a rough surface, and the filled fillers can be observed. For the AlN particle-filled SR composites (Figure 8b), the AlN fillers were observed on the fracture surface. As the AlN filler was isolated in the SR matrix, it was hard to construct a continuous structure, leading to a higher interfacial thermal resistance and lower transport of phonon. Therefore, a small improvement (8.65%) in thermal conductivity was observed for the 10%AlN/SR composites [25]. For the AlN/CNT hybrid filler-incorporated SR materials (Figure 8c–e), both granular AlN and tubular CNT were observed. With the increase in CNT loading percentage, the dispersion state and joint style of the two fillers were different. For the 8AlN+2CNT composites (Figure 8c), the AlN and CNT particles were separated from each other, and almost no continuous filler network was formed. For the 6AlN+4CNT composites (Figure 8d), obvious contact of AlN and CNT particles was observed, and some conductive networks were constructed. For the 5AlN+5CNT composites (Figure 8e), overlapping joints of AlN and CNT particles appeared, and plenty of continuous hybrid filler networks were generated, revealing the strongest synergistic effect of 5AlN+5CNT hybrid fillers [32,39,42]. Thus, the thermal conductivity of the AlN/CNT/SR composites was enhanced with the increase in CNT content, which demonstrated the above proposed thermal conductive mechanism of the AlN/CNT/SR composites. For the CNT-filled SR composites (Figure 8f), the CNT fillers were also observed to be embedded into the fracture surface. As the tubular CNTs easily formed a network within the SR matrix, relatively higher enhancement (24.04%) of thermal conductivity was observed for the 5CNT composites than for the 10%AlN/SR [54]. In addition, comparing the dispersion state of CNTs in the 5CNT composites, the dispersion/exfoliation state of CNTs in the 5AlN+5CNT composites was improved with the introduction of AlN particles. This is the other synergistic effect of hybrid fillers [23], except for the construction of a continuous filler structure.

#### 2.4.2. Thermogravimetric Analysis

**Figure 9:**
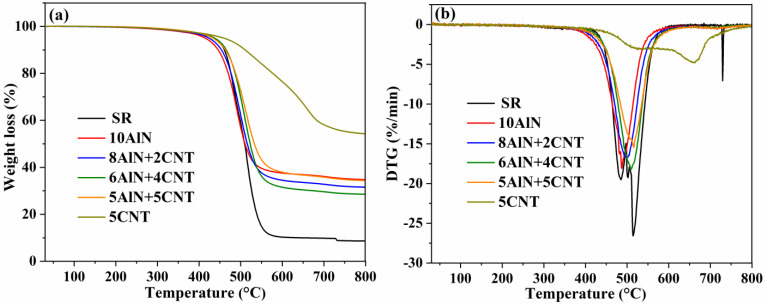
TGA (**a**) and DTGA (**b**) curves of AlN/CNT/SR nanocomposites.

As electronic packaging materials are always used at high temperatures for a very long time, such as lithium batteries and light-emitting diodes, the investigation of the thermal decomposition behavior of the obtained AlN/CNT/SR composites is very important for practical applications [6,17]. Thermogravimetric analysis (TGA) was performed to evaluate the decomposition behavior of pure SR and different SR composites, and the results are shown in Figure 9.

For the thermal decomposition curve of raw SR (Figure 9a), no obvious decomposition was observed before 300 °C, revealing that the SR was a polymeric material with high thermal stability. Fast degradation occurred after 400 °C, and the weight of residue was constant after 600 °C. The residue of SR at 800 °C was 8.6%, which was much lower than all of the other composites. Though the AlN/SR and AlN/CNT/SR composites exhibited a similar degradation behavior to pure SR, with the incorporation of ten percent AlN or AlN/CNT hybrid fillers, the residue increased to 28.6–34.8%. This can not only be ascribed to the high thermal stable of the AlN and CNT fillers but also due to the barrier effect of the filler [27,46]. The CNT/SR nanocomposites exhibited the highest thermal stability, with the residue reaching as high as 54.3%. This was due to the high specific surface area of the CNTs and the lack of a large free volume formed by micron-sized granular AlN. The residue increase in the obtained composites demonstrated the enhancement in the thermal stability with the incorporation of different fillers [53,55]. The rate of weight loss (DTG) for AlN/SR, AlN/CNT/SR, and CNT/SR composites is presented in Figure 9b. With the incorporation of AlN, AlN/CNT, and CNT fillers, the temperature for the highest rate of weight loss increased gradually, which also demonstrates the enhancement in the thermal stability. This can be attributed to the barrier effect between the SR matrix and different fillers, which can prevent the diffusion of produced volatility [32,56,57]. As the AlN and CNT fillers were used without modification, a certain degree of phase separation was observed for the obtained composites; thus, the initial decomposition temperature of the obtained composites was lower than that of the AlN/CNT incorporated poly(phenylene sulfide) [28] and SiC/Si_3_N_4_ incorporated SR [29].

The initial degradation temperature (T_5%_), the highest rate of mass loss temperature (T_H_), and the residue percentage at 800 °C (R_800_) are summarized in Table 1 based on the results of TGA and DTG. Compared with pure SR, the composites containing a low CNT loading percentage had a lower initial degradation temperature. This can be attributed to granular AlN experiencing difficulty in forming a tortuous path effect, which can prevent the diffusion of produced volatility. In addition, the T_H_ and R_800_ of all composites improved more than those of pure SR, revealing that the obtained composites exhibited higher thermal stability than that of pure SR [31,57].

For the application of SR-based materials in electronic packaging, the mechanical properties, electronic insulation, and thermal conductivity and thermal stability should be taken into account. Based on the properties of the obtained composites mentioned above, the composite containing 6AlN+4CNT hybrid fillers should be the best one, as it demonstrated the overall optimal performance. Though the 5AlN+5CNT composite had a higher thermal conductivity and thermal stability, the tensile strength, elongation at break, and surface resistivity were relatively low.

### 2.5. Comparison Study of Hybrid Filler Incorporation

The comparative evaluation of CNT-based hybrid filler-incorporated composites was summarized and is shown in Table 2. According to the reported work, it can be concluded that the AlN/CNT hybrid fillers have been widely used in the field of microelectronic devices, engineering plastic, electronic packages, microelectronics, etc. AlN/CNT hybrid filler-incorporated epoxy [27,29,32], poly(phenylene sulfide) [28], poly(L-lactide) [30], cyanate ester [31], and SR [42] exhibit much higher thermal conductivity than the AlN/CNT/SR composites in this work, which can be ascribed to the high loading percentage and chemical modification. In the reported work, the total addition of AlN/CNT hybrid fillers ranged from 20% to 73% in weight or volume, which can form plenty of conductive channels. On the other hand, chemical modification of AlN and CNTs was conducted to reduce the interface thermal resistance in all reported work. What is more, continuous networks of the prepared AlN/CNT were directly added to the polymer in some cases [28,29]. In addition, there are very few reports about the mechanical properties and electrical properties of AlN/CNT-incorporated polymer composites.

Within this work, AlN/CNT/SR composites were obtained with commercial AlN and CNT fillers, which avoided complicated chemical modification. The 6 wt.%AlN/4 wt.%CNT/SR composite had the optimal comprehensive performance, which is similar to 5 wt.%Al_2_O_3_/5 wt.%CNT incorporated SR [40]. SR-based materials with Al_2_O_3_/CNT [36,41], Al_2_O_3_/graphene [33,34,37], BN/Ag [38], CNT/Ag [39], and SiC/Si_3_N_4_ [35] hybrid filler incorporation has also been reported and showed high mechanical strength and electronic resistivity, and very high thermal conductivity. This is also due to the high loading percentage and chemical modification of the corresponding hybrid fillers.

## 3. Materials and Methods

### 3.1. Materials

The aluminum nitride (AlN, 99.9%) and carbon nanotubes (CNTs, 99.5%) were purchased from Macklin Co., Ltd. (Shanghai, China). The two-component additive liquid silicone rubber (SR, HM-906), the viscosity of component A (containing the Si-H functional group) and B (containing the Si-C=C functional group and a Pt complex catalyst) were 3600 mPa.s and 6800 mPa.s, and the curing ratio (A/B is 1:1) was provided by Haoming Topu New materials Technology Co., Ltd. (Foshan, China).

### 3.2. Preparation of AlN/CNT/SR Nanocomposites

**Figure 10:**
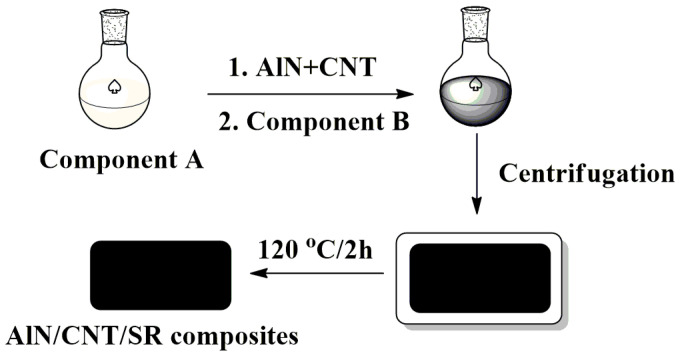
The preparation procedure of the AlN/CNT/SR composites.

The AlN/CNT/SR composites were fabricated via a thermal curing technique (Figure 10) with different AlN/CNT ratios (8/2, 6/6, 5/5 are labeled as 8AlN+2CNT, 6AlN+4CNT, 5AlN+5CNT) (Table 3) [6,58,59,60]. Typically, for the preparation of 8AlN+2CNT-incorporated composites, 0.48 g AlN and 0.12 g CNT were added to 3.0 g of low-viscosity component A, and the mixture was stirred for 1 h and sonicated for 1 h under 50 °C. Then, 3.0 g of high-viscosity component B were added and stirred for another hour. Finally, the mixture was centrifugated at 4000 r/min for 10 min to remove bubbles, poured into a polytetrafluoroethylene mold, and cured at 120 °C for 2 h. For comparison, pure silicone, 10 wt.%AlN/SR, and 5 wt.%CNT/SR composites (labeled as SR, 10AlN, and 5CNT) were also prepared under the same conditions.

### 3.3. Characterization

Scanning Electron Microscopy (SEM, ZEISS Gemini 300) from Germany Jena was used to observe the particle morphology of AlN. The surface of the AlN particles was coated with a thin gold layer, and the acceleration voltage was 20 kV. To reveal the distribution state of different fillers, the fractured surface of different SR nanocomposites was also revealed by the SEM. The morphology and size of CNTs were characterized with Philips TECNAI Transmission Electron Microscopy from Amsterdam, the Netherlands, with an acceleration voltage of 200 kV. The CNT sample was prepared by sonication in ethanol and evaporated from the suspension onto a carbon grid. The tensile strength and elongation at break of pure SR and different SR nanocomposites were determined according to GB/T 528-2009 using a Shimadzu AG-IC tensile texting machine from Tokyo, Japan, and three pieces of each sample were tested to obtain average values. The hardness of pure SR and SR nanocomposites with different fillers incorporated was measured according to GB/T 39693.9-2021 with a LX-A Shore A hardness tester from Haoxinda Instrument Co., Ltd., Shenzhen, China The surface resistivity of the obtained AlN/CNT/SR nanocomposites was determined according to GB/T 1410-2006 using an ST2643 ultra-high resistance tester from Jingge Electronic Co., Ltd., Suzhou, China. The thermal decomposition behavior of the AlN/CNT/SR nanocomposites was investigated according to GB/T 27761-2011 using a PerkinElmer TG-209-F3 thermogravimetric analyzer from Boston, MA, USA, with a heating rate of 10 °C/min from room temperature to 800 °C. The thermal conductivity of the fabricated AlN/CNT/SR nanocomposites was measured according to GB/T 11205-2009, with a DRE-2C thermal test instrument from Xiangtan Instrument Co., Ltd., Xiangtan, China.

## 4. Conclusions

In summary, to evaluate the synergistic effect of AlN and CNTs on the comprehensive properties of SR composites, AlN, CNT, and AlN/CNT hybrid fillers with different ratios of incorporated SR composites were fabricated through the thermal curing method. The synergistic effect of AlN/CNT hybrid fillers on the hardness, tensile strength, electronic insulating, thermal conductivity, and thermal stability was investigated in detail. The comprehensive properties of the obtained AlN/CNT/SR composites were better than those of the AlN/SR and CNT/SR composites. Compared with pure SR, the Shore A hardness of the 5AlN+5CNT composites increased from 58 to 63, the tensile strength of the 6AlN+4CN composites increased from 1.60 to 2.25 MPa, and the elongation at break of the 8AlN+2CN composites increased by 25.97%. With the incorporation of 5AlN+5CNT, the surface resistivity decreased from 1.96 × 10^14^ Ω/☐ to 1.20 × 10^12^ Ω/☐ through the hopping mechanism of isolated filler particles. The highest thermal conductivity of 0.162 W/m·K was observed for the 5AlN+5CNT composites, which was much higher than that of the 10AlN and 5CNT composites, revealing the synergistic effect of AlN/CNT hybrid fillers. In addition, the synergistic thermal conductive mechanism of AlN/CNT hybrid fillers was proposed and demonstrated with the fracture surface morphology of the obtained composites. Finally, the thermal stability of the obtained AlN/CNT/SR composites was investigated, and a synergistic improvement in AlN/CNT hybrid fillers was also observed. This finding will provide a basis for screening AlN/CNT hybrid fillers for electronics packaging materials, where good mechanical properties, high thermal conductivity, high thermal stability, and insulated materials are needed.

## Figures and Tables

**Table 1:** Degradation parameters of AlN/CNT/SR nanocomposites.

Sample	T_5%_ (°C)	T_H_ (°C)	R_800_ (%)
SR	436.4	485.4	8.6
10AlN	414.6	486.6	34.8
8AlN+2CNT	425.0	499.0	28.6
6AlN+4CNT	433.9	508.6	31.6
5AlN+5CNT	438.6	515.9	34.3
5CNT	458.8	661.8	54.3

**Table 2:** Comparison study of CNT-based hybrid filler-incorporated composites.

Hybrid Fillers	Parameters	Polymer (TC)	Preparation Method	Properties			Application	Ref.
				Mechanical	Electrical	Thermal		
25 vol.%AlN/1 vol.%CNT	Zirconate-coupling Agent-modified AlN, GMA-grafted CNT	EP (~0.3)	Thermal curing	/	/	1.21 W/m·K	Microelectronic devices	[27]
20 wt.%AlN/1 wt.%CNT	GPTMS-grafted AlN-g-CNT	PPS (0.28)	Melt blending	/	/	0.85 W/m·K, T_5%_ 472 °C	Engineering plastic	[28]
57.4 vol.%AlN/2 wt.%CNT	APTES-grafted AlN-g-CNT	EP (~0.2)	Thermal curing	/	/	6.25 W/m·K	Electronic packages	[29]
50 wt.%AlN/3 wt.%CNT	AlN: 1 μm, PEG-grafted CNT	PLA (0.1967)	Solution blending	/	/	0.773 W/m·K,	Electronic devices	[30]
47.5 wt.%AlN/2.5 wt.%CNT	KH-550-grafted AlN, E51-modified CNT	CE (~0.4)	Thermal curing	/	Dielectric constants (~5)	~2.3 W/m·K,	Microelectronics	[31]
3.4 vol.%AlN/0.6 vol.%CNT	Commercial, AlN: 0.5 μm, CNT: Φ 10 nm, length 20–30 μm	EP(~0.25)	Thermal curing	/	1.8–2.6 × 10^12^ Ω·m	0.53 W/m·K, T_5%_ 368 °C	Microelectronics	[32]
70 wt.%AlN/3 wt.%CNT	Ethylenediamine-grafted CNT	SR (~0.15)	Thermal curing (addition)	/	/	3.81 W/m·K	Electronic devices	[42]
6 wt.%AlN/4 wt.%CNT	Commercial, AlN: 2–5 μm, CNT: Φ 30 nm, length 1–5 μm	SR (0.104)	Thermal curing (addition)	Shore 61 A, 2.25 Mpa, elongation 88%	51 × 10^12^ Ω/☐	0.162 W/m·K, T_5%_ 434 °C, R_800_ 31.6%	Electronic packaging	This work
5 wt.%Al_2_O_3_/5 wt.%CNT	KH-304-grafted Al_2_O_3_	SR (~0.13)	Thermal curing (addition)	Shore 28 A	/	0.26 W/m·K	Electronic packaging	[40]
36 vol.%Al_2_O_3_/2 wt.%CNT	APTES-grafted Al_2_O_3_, CNT electrostatic self-assembly Al_2_O_3_	SR (~0.2)	Vulcanization (peroxide)	2.5 Mpa, 50%	1.323 × 10^9^ Ω·cm	1.137 W/m·K	Electronic packaging	[36]
20 vol.%Al_2_O_3_/0.5 wt.%CNT	Sintering Al_2_O_3_ to form a branched one	SR (~0.18)	Vulcanization (peroxide)	4.47 MPa, 206.9%	1.1 × 10^15^ Ω·cm	1.307 W/m·K	Electronic packaging	[41]
71.5 wt.%Al_2_O_3_/0.5 wt.%graphene	Graphene: 1–3 layers, 7–12 μm	Silicone grease	Blending	/	/	4.38 W/m·K	Thermal interface materials	[33]
89 wt.%Al_2_O_3_/1 wt.%graphene	Al_2_O_3_: 5 μm, Graphene: RGO	SR (~0.2)	Vulcanization (peroxide)	~0.3 MPa	/	~3.4 W/m·K	Heat dissipation	[34]
63 vol.%Al_2_O_3_/1 wt.%graphene	Al_2_O_3_: 0.7 μm, Graphene: RGO	Silicone grease	Blending	/	/	3.45 W/m·K	Thermal interface materials	[37]
30 vol.%BN-Ag	PDA-grafted BN-g-Ag	SR (~0.2)	Vulcanization (peroxide)	~2.0 Mpa, elongation 50%	1.89 × 10^−11^ S/cm	0.75 W/m·K	Electronic equipment	[38]
30 vol.%CNT-Ag	PDA -rafted CNT-g-Ag	SR (~0.2)	Vulcanization (peroxide)	/	/	0.655 W/m·K	Electronic equipment	[38]
5 vol.%SiC/45 vol.%Si_3_N_4_	SiC: 2 μm, Si_3_N_4_: Φ 100 nm, length 20 μm	SR (~0.2)	Vulcanization (peroxide)	2.81 Mpa	/	1.48 W/m·K, T_5%_ 551 °C	Heat dissipation	[35]

**Table 3:** Compositions of the AlN/CNT/SR nanocomposites.

Sample	AlN (g)	CNT (g)
SR	-	-
10AlN	0.60	-
8AlN+2CNT	0.48	0.12
6AlN+4CNT	0.36	0.24
5AlN+5CNT	0.30	0.30
5CNT	-	0.30

## Data Availability

Data produced in this study can be made available upon a reasonable request.
